# Risk of Raynaud's Phenomenon Among Workers in the Occupational Disease Surveillance System

**DOI:** 10.1002/ajim.23700

**Published:** 2025-01-09

**Authors:** Ryann E. Yeo, Fanni R. Eros, Paul A. Demers, Jeavana Sritharan

**Affiliations:** ^1^ Occupational Cancer Research Centre, Ontario Health Toronto Ontario Canada; ^2^ Dalla Lana School of Public Health University of Toronto Toronto Ontario Canada

**Keywords:** occupation, Raynaud's phenomenon, sex differences, surveillance, vibration white finger

## Abstract

**Introduction:**

Raynaud's phenomenon (RP) is linked to occupational exposures such as vibration, cold temperature, and chemicals. However, large cohort studies examining RP by occupation and sex are scarce. To address this gap, this study aimed to assess risk of RP by both occupation and sex in a large cohort of workers in Ontario, Canada.

**Methods:**

Workers with accepted lost‐time compensation claims were linked to physician billing records to identify diagnoses of RP between 2002 and 2020. A 3‐year washout (disease‐free) period was applied, and follow‐up was limited to 5 years. Cox proportional hazard models were used to estimate hazard ratios (HR) and 95% confidence intervals (CI) for diagnoses of RP, adjusted for age at start of follow‐up, birth year, and stratified by sex.

**Results:**

A total of 7,131 RP cases were identified among 810,739 workers. Among men, higher risks were observed for truck drivers (HR = 1.23, 95% CI = 1.08–1.41), driver‐salesmen (HR = 2.54, 95% CI = 1.21–5.34), those in mining and quarrying‐related cutting, handling, and loading (HR = 2.57, 95% CI = 1.29–5.15), and construction trades laboring and elemental work (HR = 1.70, 95% CI = 1.24–2.34). Among women, higher risks were observed for those working in waitressing and related (HR = 1.70, 95% CI = 1.22–2.38), food and beverage preparation (HR = 1.34, 95% CI = 1.02–1.76), and electrical equipment fabricating and assembling (HR 1.96, 95% CI = 1.08–3.55).

**Conclusion:**

Study findings show elevated risks of RP among various occupations, with notable differences between men and women. These differences may be attributable to variations in potential exposures and susceptibility to RP. Findings underscore the need for large cohort studies to examine RP across various occupational groups and both sexes.

## 1 Introduction

Raynaud's phenomenon (RP) affects around 3%–5% of the general population [1]. It is a condition that causes blood vessels in the extremities to narrow and restrict blood flow, resulting in pale, white, and blue fingers, or toes. This phenomenon can lead to numbness, tingling, and pain which can interfere with daily activities [2]. Risk factors for RP include certain medications (e.g., β‐adrenoceptor blockers, clonidine), age (younger age for females, and older age for males), genetic history, cigarette smoking, alcohol consumption, anxiety, and stress [1, 3, 4, 5]. Most cases are referred to as primary RP (PRP), with no known cause, while others are referred to as secondary RP (SRP), which is linked to conditions such as lupus or scleroderma, or work‐related exposures [2]. PRP accounts for 85% of cases in women, with 15% being SRP, whereas in men, the distribution between primary and secondary is more even [6]. Additionally, women are more likely to experience an earlier onset and have a family history of RP [1].

In Canada, the majority of occupation‐related studies on RP are historical, cross‐sectional in design, and often focus on small workplace cohorts [7, 8, 9, 10, 11]. Previous studies have also relied on self‐reporting of symptoms related to RP [12, 13]. Occupation‐related RP would be considered secondary RP, which can manifest more severe symptoms than PRP, including small, painful sores, and in rare cases, skin ulcers and gangrene [2]. The mechanism by which exposures or conditions in occupational settings can lead to RP remains elusive. However, it is thought to be linked to vibration exposure [14], cold temperatures [3, 15], emotional stress [3], and specific chemicals such as organic solvents and vinyl chloride [16, 17, 18].

Vibration‐specific RP, also known as vibration‐induced white finger (VWF) or hand‐arm vibration syndrome (HAVS), is a form of SRP associated with occupational vibration exposure [9, 10, 13, 14, 19, 20]. Vibration exposure primarily occurs through the use of power or pneumatic hand tools, as well as machinery used in forestry, transportation, construction, or agriculture [19]. Vibrations can be transmitted through various touch points such as the hands, feet, back, and buttocks, either individually or in combination [7, 14]. Several cross‐sectional studies have investigated the prevalence of SRP in specific occupations, such as forestry [8, 21], mining [7, 9, 22, 23], and rock drilling [10]. For some individuals, reversal of RP symptoms can occur with reduction or cessation of vibration exposure [24].

RP is often triggered through cold exposure which can occur through the environment or localized handling of cold objects [25]. The resultant finger blanching after cold exposure or cold provocation test is one of the main criteria for being diagnosed with RP [2, 26, 27, 28]. Working in colder‐than‐normal environments may also increase the risk of RP. Cold exposure through weather conditions or cold climates may be a risk, as is demonstrated in the elevated RP prevalence for electricians working in the winter versus summer months [29], or the higher prevalence of vibration‐related SRP in colder climates [30, 31]. Furthermore, a cold work environment may also increase risk (e.g., in food processing occupations) [32]. A study in northern Sweden, where cold exposure is common during work and leisure time, identified cumulative cold exposure associated with self‐reporting of RP, further demonstrating the association between RP and temperature [33].

Chemical exposures may also increase the risk of SRP, specifically organic solvents, and with limited evidence, vinyl chloride monomer [18, 34, 35, 36]. Workers may be exposed to vinyl chloride in polyvinyl chloride manufacturing plants [16, 37]. Organic solvent exposure is likely to occur in laboratories, printing, and painting, among other occupations [17, 18]. However, over the past few decades, workplace regulations have improved to limit worker exposure to these chemicals [38, 39].

There is also some evidence linking emotional stress to risk of RP. Increased stress ratings triggered both PRP and SRP onset in one‐third of participants in a randomized control trial in the United States [3]. Similarly, in a cohort study in France, high psychological demand and low supervisor support significantly increased the odds of RP [25]. Additionally, a study on food processing workers in France identified work‐related factors such as fewer rest breaks, unheated break areas, repetitive movements, and arm or hand exertion as contributing to the risk of RP, further suggesting that multiple factors may be involved [30].

Currently, there are no large cohort studies that have been able to examine the risk of RP across many occupational groups or by sex. To address this gap, this study used an existing cohort of workers to investigate the sex‐specific risk of RP by occupation.

## 2 Methods

### 2.1 Study Cohort and Data Sources

This study used the Occupational Disease Surveillance System (ODSS), a large cohort of workers established through the linkage of various administrative datasets in Ontario.

Briefly, workers were first identified in the Ontario Workplace Safety and Insurance Board (WSIB) accepted lost time compensation claims records (1983–2019). For this analysis, workers were eligible for inclusion if they had a non‐RP‐related compensation claim with occupation information between January 1, 2002, and December 31, 2019 (*n* = 905,578). Workers with a WSIB claim for RP were considered prevalent cases and, therefore, were not included. If a worker had more than one claim but the claims were in the same occupation, then only the first claim was used. If the claims were in different occupations, then all claims for different occupations were included. Less than 10% of the cohort had more than one claim in different occupations. Workers were then linked to the Ontario Health Insurance Plan's Registered Persons Database (RPDB), which contains information on death, emigration, residence, and the provincial Health Insurance Number (HIN). Workers who emigrated out of Ontario or died before cohort entry were removed from the analysis (*n* = 2490), as identified in the RPDB. Workers who did not have a HIN were also removed (n = 76,738). Using the HIN, a total of 826,350 workers were successfully linked to the Ontario Health Insurance Plan eClaims database (OHIP, physician billing records). Further, only workers aged 15–65 years were included to reflect the working‐age population (*n* = 813,835). Details of the linkage process are described elsewhere [40].

### 2.2 Exposure

Occupation information was obtained from the compensation claims records and coded by the WSIB using the Canadian Classification Dictionary of Occupations (CCDO 1971). Occupation was classified into three levels: division, major, and minor groups [40]. Division groups represent the broadest level of classification, major groups represent the intermediate level of classification, and minor groups represent a detailed classification of the occupation. Potential workplace exposures within occupations may overlap across various job titles, therefore we can only base findings on job titles and speculate about potential workplace exposures within these occupations.

### 2.3 Outcome

Cases of Raynaud's phenomenon were identified using OHIP physician billing records from 1999 to 2020. Cases were defined using the International Classification of Disease (ICD) ninth revision (ICD‐9) diagnosis code 443 (“Other peripheral vascular disease”), which includes Raynaud's phenomenon and other and unspecified peripheral vascular diseases. Although this ICD code includes other conditions, RP is much more common than the other peripheral vascular diseases (Buerger's disease, other arterial dissections, and other peripheral vascular diseases, etc.) [41, 42]. The 443 ICD code also includes a more common condition, intermittent claudication, however this only refers to unspecified claudication whereas ICD code 440 includes atherosclerosis‐related claudication which makes up the majority of claudication cases and was not used in this case definition [43]. Furthermore, RP has known associations with work‐related factors, unlike the other conditions [44]. Using the OHIP physician billing records, an RP diagnosis was based on at least two visits with a physician on two separate days, within 5 years of cohort entry. There was no required lag time between diagnoses, other than the separate day requirement. The diagnostic codes for RP may include both PRP and SRP as there is no way to differentiate between the two types in diagnostic coding. Although RP cases could be identified in OHIP records as early as January 1, 1999, the earliest possible date for cohort entry was January 1, 2002, to account for a 3‐year washout (disease‐free) period. The latent period for the onset of occupational‐related RP depends on the type and severity of the exposure. There is evidence that RP symptoms can appear after 1 year of vibration exposure, with one study finding up to 70% of exposed workers developing symptoms after 3 years [45]. While less is known about the latency of RP‐related to cold or chemical exposure, the latency for a vinyl chloride‐related condition (acro‐osteolysis), which can cause RP, is 5 months to 3.5 years [46]. Therefore, a 3‐year washout period is useful in capturing as many RP cases as possible, while still removing prevalent cases. Those with an OHIP RP diagnosis date before their cohort entry were also considered prevalent cases and therefore excluded from the analysis (n = 3096).

### 2.4 Statistical Analysis

Workers were followed from their WSIB compensation claim date after 2002, until the date of first RP diagnosis, emigration out of Ontario, death, age 65, end of the 5‐year follow‐up period, or end of the study period (December 31, 2020), whichever occurred first. The restricted 5‐year follow‐up period was applied to examine occupation information closer to the diagnosis of RP. Cox proportional hazard models were used to determine the hazard ratios (HR) and 95% confidence intervals (CI) for each occupation group at the division, major and minor levels. All models were adjusted for age at the start of follow‐up (continuous) and birth year, and stratified by sex. Age at the start of follow‐up can account for changes in risk with age, while birth year may account for changes over time, such as those related to regulations, policies, and exposure. A separate model was created for each occupation. The risk of RP in each occupation was compared to all other workers in the cohort.

In accordance with the organization's disclosure guidelines, no counts less than six were reported. All analyses were performed using SAS V.9.4.

### 2.5 Ethics Approval

This study was approved by the University of Toronto Health Sciences Research Ethics Board (#39013).

## 3 Results

The cohort consisted of 810,739 workers, of which 61% were male. A total of 4713 males and 2418 females were diagnosed with RP during the follow‐up period. Both males and females with RP had an earlier median birth year and were older at the start and end of follow‐up compared to the overall cohort (Table 1).

**Table 1:** Characteristics of workers diagnosed with Raynaud's phenomenon and the overall ODSS cohort.

	RP cases (*N* = 7131)		Overall ODSS cohort (*N* = 810,739)	
	**Male (*N*** = **4713)**	**Female (*N*** = **2418)**	**Male (*N*** = **491,782)**	**Female (*N*** = **318,957)**
	**Median (IQR)**	**Median (IQR)**	**Median (IQR)**	**Median (IQR)**
Year of birth	1958 (1953, 1963)	1959 (1954, 1965)	1970 (1960, 1981)	1967 (1958, 1979)
Age at start of follow‐up	48 (42, 53)	48 (41, 53)	38 (28, 48)	42 (31, 51)
Age at end of follow‐up	53 (47, 58)	52 (46, 57)	44 (33, 54)	48 (37, 56)
Years of follow‐up	5 (3, 5)	5 (3, 5)	5 (5,5)	5 (5,5)

Abbreviations: IQR, interquartile range; ODSS, occupational disease surveillance system; RP, Raynaud's phenomenon.

### 3.1 Risk of Raynaud's Phenomenon by Broad Occupational Groups

Adjusted HRs and corresponding 95% CIs for RP by division and major level occupational group with at least 6 cases for either males or females are shown in Table 2. At the major level, hazard ratios were increased by more than 10% for both males and females employed in other clerical and related; food and beverage preparation services; other service; metal processing and related; food and beverage and related processing; metal shaping and forming (except machining); other product fabricating, assembling, and repairing; motor transport operating; material handling and related; and printing and related.

**Table 2:** Hazard ratios (HR) and 95% confidence intervals (CI) for Raynaud's phenomenon by select broad division and major‐level occupation group.

	Males (*N* = 491,782)		Females (*N* = 318,957)	
Occupation	Cases (workers)	HR^a^ (95% CI)	Cases (workers)	HR^a^ (95% CI)
*Managerial, administrative, and related*	48 (9035)	1.05 (0.78–1.39)	36 (12,436)	0.82 (0.59–1.15)
Officials and administrators unique to government	8 (1220)	1.22 (0.61–2.44)	< 6 (2090)	—
Other managers and administrators	30 (5130)	1.14 (0.80–1.64)	14 (4540)	0.89 (0.52–1.51)
Related to management and administration	11 (2810)	0.79 (0.43–1.42)	18 (6007)	0.85 (0.53–1.36)
*Natural sciences, engineering, and mathematics*	30 (9555)	0.76 (0.53–1.09)	10 (3109)	1.00 (0.54–1.87)
Architects and engineers	6 (1451)	0.80 (0.36‐1.79)	< 6 (344)	—
Other architecture and engineering	16 (5860)	0.70 (0.43–1.14)	< 6 (1416)	—
*Social sciences and related fields*	15 (3995)	1.01 (0.61–1.68)	39 (14,725)	0.88 (0.64–1.21)
Social work and related fields	14 (2827)	1.42 (0.84–2.40)	34 (11,007)	1.08 (0.77–1.52)
Other social sciences and related fields	< 6 (649)	—	6 (2568)	0.75 (0.33–1.67)
*Teaching and related*	14 (5574)	0.59 (0.35–1.01)	73 (27,413)	0.84 (0.66–1.06)
Elementary and secondary school teaching and related	9 (4699)	*0.47 (0.25–0.91)*	68 (25,504)	0.84 (0.66–1.08)
*Medicine and health*	24 (9946)	0.70 (0.47–1.05)	167 (61,435)	*0.83 (0.70–0.98)*
Nursing therapy and related assisting	24 (8519)	0.84 (0.56–1.25)	147 (53,697)	*0.82 (0.69–0.98)*
Other medicine and health	< 6 (1582)	—	27 (9875)	0.96 (0.66–1.41)
*Artistic, literary, recreational, and related*	21 (5216)	1.24 (0.81–1.91)	6 (4840)	0.59 (0.27–1.32)
Performing and audiovisual arts	6 (1152)	1.39 (0.63–3.11)	< 6 (505)	—
Sport and recreation	9 (3235)	0.98 (0.51–1.90)	< 6 (3608)	—
*Clerical and related*	179 (38,599)	1.15 (0.98–1.34)	166 (42,062)	1.16 (0.98–1.37)
Stenographic and typing	0 (82)	—	10 (2686)	0.83 (0.45–1.55)
Bookkeepers, tellers and cashiers, and other clerks	< 6 (2,238)	—	54 (12,338)	**1.37 (1.04–1.80)**
Material recording, scheduling, and distributing	112 (21,655)	**1.30 (1.08–1.58)**	19 (5456)	1.03 (0.65–1.62)
Reception, information, mail, and message distribution	36 (9311)	0.85 (0.61–1.18)	29 (7386)	1.10 (0.76–1.59)
Other clerical and related	36 (6123)	**1.46 (1.05–2.03)**	63 (14,916)	1.23 (0.95–1.59)
*Sales*	136 (37,823)	**1.19 (1.00–1.42)**	125 (40,374)	1.04 (0.86–1.25)
Sales commodities	118 (35,685)	1.10 (0.91–1.32)	122 (38,094)	1.08 (0.89–1.30)
Other sales	18 (2374)	**2.29 (1.44–3.64)**	< 6 (2633)	—
*Service*	295 (80,512)	1.06 (0.93–1.20)	273 (82,369)	1.15 (1.00–1.32)
Protective service	69 (22,443)	0.98 (0.77–1.24)	17 (6373)	1.09 (0.67–1.76)
Food and beverage preparation and related service	59 (25,134)	1.17 (0.90–1.52)	109 (35,129)	**1.25 (1.02–1.52)**
Lodging and other accommodation	6 (953)	1.50 (0.68–3.35)	< 6 (1036)	—
Personal service	6 (1983)	0.72 (0.33–1.62)	69 (20,498)	1.02 (0.80–1.30)
Apparel and furnishing service	< 6 (903)	—	9 (2049)	1.04 (0.54–2.01)
Other service	171 (31,955)	1.10 (0.94–1.29)	89 (22,118)	1.11 (0.90–1.39)
*Farming, horticultural and animal husbandry*	46 (17,740)	0.88 (0.66–1.18)	9 (5256)	0.74 (0.38–1.43)
Other farming, horticultural and animal husbandry	41 (17,308)	0.81 (0.59–1.10)	9 (5113)	0.77 (0.40–1.48)
*Forestry and logging*	14 (2202)	1.51 (0.89–2.55)	< 6 (186)	—
*Mining and quarrying including oil and gas field*	21 (2903)	**1.80 (1.17–2.77)**	< 6 (83)	—
*Processing (mineral, metal, chemical)*	112 (21,747)	1.20 (0.99–1.45)	28 (5949)	1.29 (0.89–1.88)
Metal processing and related	49 (8954)	1.22 (0.92–1.62)	7 (793)	**2.38 (1.13–5.01)**
Clay, glass, and stone processing, forming and related	17 (2288)	**1.68 (1.04–2.71)**	< 6 (167)	—
Chemicals, petroleum, rubber, plastic, and related materials processing	51 (11,116)	1.09 (0.83–1.44)	21 (5055)	1.14 (0.74–1.76)
*Processing (food, wood, textile)*	111 (24,114)	1.17 (0.96–1.42)	54 (12,733)	1.26 (0.96–1.66)
Food and beverage and related processing	68 (15,666)	1.10 (0.86–1.40)	46 (10,448)	1.33 (0.99–1.79)
Wood processing except paper pulp	9 (1829)	1.16 (0.60–2.23)	< 6 (213)	—
Pulp and papermaking and related occupations	7 (1187)	1.12 (0.53–2.34)	0 (227)	—
Textile processing	9 (1610)	1.21 (0.63–2.32)	< 6 (1143)	—
Other processing	20 (4399)	1.36 (0.88–2.11)	< 6 (833)	—
*Machining and related*	275 (52,871)	**1.19 (1.05–1.36)**	31 (6339)	1.33 (0.93–1.90)
Metal machining	58 (12,150)	0.99 (0.76–1.28)	< 6 (930)	—
Metal shaping and forming except machining	205 (38,507)	**1.24 (1.07–1.43)**	23 (4903)	1.26 (0.83–1.90)
Wood machining	15 (2840)	1.15 (0.69–1.91)	< 6 (373)	—
Other machining and related	13 (2760)	0.85 (0.49–1.46)	< 6 (377)	—
*Product fabricating, assembling and repairing*	389 (86,598)	1.04 (0.93–1.16)	87 (18,513)	**1.30 (1.04–1.62)**
Fabricating and assembling metal products, n.e.c.	92 (18,675)	1.07 (0.87–1.33)	30 (7021)	1.24 (0.86–1.78)
Fabricating, assembling, installing, and repairing electrical and electronic and related equipment	51 (11,545)	0.96 (0.72–1.26)	16 (2799)	1.36 (0.83–2.23)
Fabricating, assembling, and repairing wood products	30 (6715)	1.07 (0.74–1.53)	6 (717)	2.23 (1.00–4.98)
Fabricating, assembling, and repairing textile, fur, and leather products	8 (1536)	0.96 (0.48–1.92)	11 (2657)	0.91 (0.50–1.65)
Fabricating, assembling, and repairing rubber, plastic, and related products	13 (2024)	1.17 (0.68–2.03)	< 6 (556)	—
Mechanics and repairers except electrical	160 (38,471)	0.95 (0.81–1.12)	< 6 (1230)	—
Other product fabricating, assembling, and repairing	85 (16,131)	1.20 (0.96–1.49)	28 (5339)	1.44 (0.99–2.10)
Construction trades	317 (81,974)	1.07 (0.94–1.20)	7 (2223)	1.19 (0.57–2.51)
Excavating, grading, paving, and related	42 (6665)	1.21 (0.89–1.65)	< 6 (224)	—
Electrical power, lighting, and wire communications equipment erecting, installing, and repairing	38 (12,274)	0.75 (0.54–1.03)	0 (403)	—
Other construction trades	245 (64,715)	1.11 (0.97–1.27)	< 6 (1607)	—
*Transport equipment operating*	329 (65,108)	1.10 (0.97–1.24)	28 (7896)	1.09 (0.75–1.59)
Air transport operating	8 (4575)	0.62 (0.31–1.24)	< 6 (865)	—
Motor transport operating	284 (51,455)	**1.16 (1.02–1.32)**	16 (3329)	1.38 (0.84–2.26)
Other transport and related equipment operating	47 (10,064)	0.97 (0.73–1.30)	13 (3980)	0.98 (0.57–1.69)
*Materials handling and related, n.e.c.*	207 (45,154)	**1.24 (1.08–1.44)**	37 (9764)	1.14 (0.82–1.58)
*Other crafts and equipment operating*	36 (6277)	1.12 (0.81–1.56)	11 (1608)	1.80 (0.99–3.26)
Printing and related	21 (3638)	1.18 (0.76–1.81)	9 (1277)	1.78 (0.92–3.43)
Stationary engine and utilities equipment operating and related	14 (2303)	1.07 (0.63–1.81)	0 (154)	—

*Note:* Statistically significant increased risks are bolded, statistically significant decreased risks are italicized

Abbreviations: CI, confidence interval; HR, hazard ratio; n.e.c., not elsewhere classified.

^a^ Adjusted for age at start of follow‐up and birth year.

Associations were stronger among males in diverse occupations such as material recording, scheduling, and distributing; other sales; mining and quarrying; other construction trades; and clay, glass, and stone processing and forming. Risk in some of these occupations could not be assessed among females due to low case counts. However, stronger associations were observed among females employed as bookkeepers, tellers and cashiers and other clerks; and wood product fabricating, assembling, and repairing. There were reduced risks for males in elementary and secondary school teaching and for females in nursing therapy and related within medicine and health (Table 2).

### 3.2 Risk of Raynaud's Phenomenon by Specific Occupational Groups

Table 3 presents the results for specific minor level occupational groups with at least 10 cases for either males of females or a significant HR. Both males and females employed in various specific occupational groups demonstrated a hazard ratio greater than 10%. This included males and females employed as stock clerks; other clerical and related; guards and watchmen; food and beverage waiters, hostesses, and stewards; slaughtering and meat cutting, canning, curing, and packing; laboring and other elemental work in food, beverage, and related processing; metalworking‐machine operators; motor vehicle fabricating and assembling; other product fabricating assembling, and repairing; truck drivers; and laboring and other elemental work in material handling occupations.

**Table 3:** Hazard ratios (HR) and 95% confidence intervals (CI) for Raynaud's phenomenon by select minor‐level occupation groups.

	Males (*N* = 491,782)		Females (*N* = 318,957)	
Occupation	Cases (Workers)	HR^a^ (95% CI)	Cases (Workers)	HR^a^ (95% CI)
*Managerial, administrative, and related*				
Other managers and administrators, n.e.c.	12 (1921)	1.21 (0.68–2.13)	< 6 (1244)	—
Management and administration related, n.e.c.	< 6 (892)	—	12 (3593)	0.93 (0.52–1.64)
*Natural sciences, engineering, and mathematics*				
Architectural and engineering technologists and technicians	13 (5035)	0.68 (0.39–1.17)	< 6 (1302)	—
*Social sciences and related fields*				
Social workers	< 6 (596)	—	10 (2375)	1.29 (0.69–2.40)
Welfare and community services	12 (2422)	1.46 (0.83–2.58)	23 (9066)	0.91 (0.60–1.38)
*Teaching and related*				
Elementary and kindergarten teachers	< 6 (2261)	—	34 (12,542)	0.86 (0.61–1.22)
Secondary school teachers	< 6 (1646)	—	14 (3306)	1.28 (0.76–2.17)
Elementary and secondary school teaching and related, n.e.c.	< 6 (1069)	—	28 (11,173)	0.79 (0.54–1.15)
*Medicine and health*				
Nurses, registered, graduate and nurses‐in‐training	< 6 (1257)	—	42 (17,066)	*0.72 (0.53–0.99)*
Nursing assistants	< 6 (651)	—	23 (7189)	0.91 (0.60–1.37)
Nursing aides and orderlies	12 (2801)	1.01 (0.57–1.79)	80 (26,401)	0.92 (0.73–1.16)
Nursing, therapy, and related assisting, n.e.c.	8 (4189)	0.67 (0.34–1.35)	12 (6129)	0.61 (0.35–1.09)
Medical laboratory technologists and technicians	< 6 (925)	—	23 (6994)	1.21 (0.80–1.84)
*Clerical and related*				
Tellers and cashiers	< 6 (1242)	—	42 (9330)	**1.52 (1.12–2.07)**
Shipping and receiving clerks	80 (14,906)	**1.40 (1.12–1.76)**	8 (2579)	0.94 (0.47–1.89)
Stock clerks and related	29 (4624)	1.39 (0.97–2.01)	7 (1756)	1.13 (0.54–2.38)
General office clerks	< 6 (565)	—	15 (4486)	0.80 (0.48–1.33)
Mail carriers	20 (4491)	0.93 (0.60–1.44)	9 (3026)	0.93 (0.48–1.80)
Mail and postal clerks	10 (2465)	0.84 (0.45–1.57)	11 (2381)	1.15 (0.64–2.09)
Messengers	11 (2997)	0.85 (0.47–1.55)	< 6 (697)	—
Other clerical and related, n.e.c.	24 (4196)	1.48 (0.99–2.21)	39 (8305)	**1.49 (1.08–2.06)**
*Sales*				
Supervisors: sales, commodities	19 (5284)	0.84 (0.53–1.32)	20 (7982)	0.82 (0.53–1.28)
Salesmen and salespersons, commodities, n.e.c.	85 (28415)	1.08 (0.87–1.34)	99 (29,619)	1.14 (0.93–1.40)
Sales clerks, commodities	11 (2187)	1.13 (0.63–2.05)	6 (1721)	0.78 (0.35–1.75)
Driver‐salesmen	7 (388)	**2.54 (1.21–5.34)**	0 (4)	—
Other sales, n.e.c.	7 (909)	**2.90 (1.38–6.08)**	< 6 (1070)	—
*Service*				
Fire‐fighting	22 (5140)	1.20 (0.78–1.82)	< 6 (368)	—
Policemen and detectives, government	12 (8864)	*0.51 (0.29–0.90)*	< 6 (2226)	—
Guards and watchmen	35 (8571)	1.20 (0.86–1.68)	11 (3672)	1.14 (0.63–2.06)
Chefs and cooks	29 (12,456)	1.19 (0.82–1.71)	21 (7379)	0.96 (0.62–1.47)
Waiters, hostesses and stewards, food and beverage	11 (2357)	1.77 (0.98–3.19)	36 (9297)	**1.70 (1.22–2.38)**
Food and beverage preparation and related service, n.e.c.	14 (8976)	0.83 (0.49–1.41)	55 (15,777)	**1.34 (1.02–1.76)**
Personal service, n.e.c.	< 6 (1459)	—	63 (17,682)	1.04 (0.80–1.34)
Janitors, charworkers, and cleaners	149 (24,860)	1.15 (0.97–1.36)	77 (19,547)	1.08 (0.85–1.36)
Laboring and other elemental work in services	14 (4803)	0.86 (0.51–1.45)	11 (1727)	1.73 (0.95–3.13)
*Farming, horticultural and animal husbandry*				
Farm workers	12 (6221)	0.60 (0.34–1.06)	< 6 (1760)	—
Nursery and related workers	29 (9924)	1.09 (0.75–1.57)	< 6 (2071)	—
*Mining and quarrying including oil and gas field*				
Mining and quarrying: cutting, handling, and loading	8 (638)	**2.57 (1.29–5.15)**	< 6 (22)	—
Mining and quarrying including oil and gas field, n.e.c.	11 (1732)	1.64 (0.90–2.96)	0 (29)	—
*Processing (mineral, metal, chemical)*				
Moulding, coremaking, and metal casting	12 (2030)	1.25 (0.71–2.20)	< 6 (160)	—
Metal processing and related, n.e.c.	25 (5391)	1.11 (0.75–1.64)	< 6 (477)	—
Clay, glass, and stone processing, forming and related, n.e.c.	6 (393)	**2.53 (1.13–5.63)**	< 6 (53)	—
Laboring and other elemental work in chemicals, petroleum, rubber, plastic, and related materials processing	10 (2987)	0.92 (0.49–1.71)	10 (1402)	**2.11 (1.13–3.94)**
Chemicals, petroleum, rubber, plastic, and related materials processing, n.e.c.	37 (7845)	1.10 (0.79–1.52)	12 (3709)	0.86 (0.49–1.52)
*Processing (food, wood, textile)*				
Baking, confectionery making, and related	< 6 (1765)	—	10 (2177)	1.56 (0.84–2.90)
Slaughtering and meat cutting, canning, curing, and packing	16 (3261)	1.19 (0.73–1.95)	8 (1003)	**2.46 (1.23–4.93)**
Laboring and other elemental work in food, beverage, and related processing	35 (7736)	1.21 (0.86–1.69)	22 (5667)	1.11 (0.73–1.70)
Food, beverage, and related processing, n.e.c.	10 (3007)	0.73 (0.39–1.36)	6 (1516)	1.06 (0.47–2.36)
Laboring and other elemental work in other processing	20 (4313)	1.40 (0.90–2.18)	< 6 (784)	—
*Machining and related*				
Tool and die making	10 (3117)	0.73 (0.39–1.35)	0 (139)	—
Machinist and machine tool setting‐up	40 (6052)	1.28 (0.94–1.75)	< 6 (410)	—
Machine tool operating	13 (3500)	0.77 (0.45–1.34)	< 6 (360)	—
Forging	10 (943)	**2.05 (1.10–3.82)**	< 6 (81)	—
Sheet metal workers	19 (2832)	1.41 (0.90–2.22)	< 6 (127)	—
Metalworking‐machine operators, n.e.c.	85 (14,810)	1.16 (0.93–1.44)	17 (3079)	1.39 (0.86–2.25)
Welding and flame cutting	76 (14,594)	1.25 (0.99–1.57)	< 6 (800)	—
Boilermakers, platers, and structural metal workers	11 (2003)	1.09 (0.60–1.98)	0 (160)	—
Metal shaping and forming, except machining, n.e.c.	29 (6720)	1.18 (0.82–1.71)	< 6 (877)	—
Planning, turning, shaping, and related wood machining	11 (2383)	1.04 (0.57–1.88)	< 6 (328)	—
*Product fabricating, assembling, and repairing*				
Motor vehicle fabricating and assembling, n.e.c.	62 (13,350)	1.11 (0.86–1.43)	26 (5849)	1.35 (0.92–2.00)
Industrial, farm, construction, and other mechanized equipment and machinery fabricating and assembling, n.e.c.	10 (2335)	0.91 (0.49‐1.69)	< 6 (598)	—
Other fabricating and assembling, metal products, n.e.c.	15 (1936)	1.25 (0.75–2.08)	< 6 (615)	—
Electrical equipment fabricating and assembling	16 (2867)	1.05 (0.64–1.72)	11 (1288)	**1.96 (1.08–3.55)**
Electrical and related equipment installing and repairing, n.e.c.	12 (4491)	0.65 (0.37–1.15)	< 6 (157)	—
Electronic and related equipment installing and repairing, n.e.c.	15 (3151)	1.10 (0.66–1.83)	< 6 (1083)	—
Cabinet and wood furniture makers	20 (3550)	1.29 (0.83–2.00)	< 6 (379)	—
Motor vehicle mechanics and repairmen	92 (23,496)	1.08 (0.88–1.34)	< 6 (686)	—
Industrial, farm, and construction machinery mechanics and repairmen	59 (13,189)	0.78 (0.60–1.02)	0 (316)	—
Mechanics and repairmen, except electrical, n.e.c.	18 (2754)	0.96 (0.60–1.52)	0 (133)	—
Painting and decorating, except construction	21 (3274)	1.27 (0.83–1.96)	< 6 (514)	—
Other product fabricating, assembling, and repairing, n.e.c.	50 (10,561)	1.16 (0.88–1.54)	26 (4266)	**1.69 (1.14–2.49)**
*Construction trades*				
Excavating, grading, and related	26 (4626)	1.12 (0.76–1.65)	< 6 (157)	—
Excavating, grading, paving, and related, n.e.c.	10 (458)	**2.72 (1.46–5.06)**	0 (14)	—
Construction electricians and repairmen	24 (6679)	0.84 (0.56–1.26)	0 (122)	—
Foremen: other construction trades	14 (2724)	1.05 (0.62–1.78)	0 (77)	—
Carpenters and related	38 (10,485)	0.96 (0.70–1.33)	0 (144)	—
Painters, paperhangers, and related	16 (2301)	1.44 (0.88–2.36)	0 (228)	—
Roofing, waterproofing, and related	10 (2990)	1.30 (0.70–2.43)	0 (27)	—
Pipefitting, plumbing, and related	35 (6300)	1.36 (0.97–1.90)	< 6 (146)	—
Laboring and other elemental work in other construction trades	39 (4328)	**1.70 (1.24–2.34)**	0 (35)	—
Other construction trades, n.e.c.	99 (32,568)	1.04 (0.85–1.27)	< 6 (778)	—
*Transport equipment operating*				
Truck drivers	258 (42,780)	**1.23 (1.08–1.41)**	11 (2054)	1.52 (0.84–2.76)
Motor transport operating, n.e.c.	37 (10,267)	0.92 (0.66–1.27)	< 6 (888)	—
Other transport and related equipment operating, n.e.c.	47 (10,023)	0.98 (0.73–1.31)	13 (3979)	0.98 (0.57–1.69)
*Materials handling and related, n.e.c.*				
Hoisting, n.e.c.	13 (1438)	1.69 (0.98–2.91)	0 (35)	—
Longshoremen, stevedores, and freight handlers	30 (5857)	1.07 (0.74–1.53)	0 (335)	—
Materials handling equipment operators, n.e.c.	19 (1949)	1.47 (0.94–2.32)	0 (73)	—
Packaging, n.e.c.	11 (1708)	1.26 (0.70–2.29)	6 (1352)	0.93 (0.42–2.07)
Laboring and other elemental work in materials handling	142 (35,813)	1.18 (0.99–1.40)	32 (8093)	1.30 (0.92–1.85)
Materials handling and related, n.e.c.	13 (1767)	1.31 (0.76–2.27)	< 6 (175)	—
*Other crafts and equipment operating*				
Printing press	11 (1871)	1.13 (0.62–2.04)	< 6 (278)	—
Printing and related, n.e.c.	10 (1001)	**2.06 (1.11–3.82)**	< 6 (477)	—
Stationary engine and utilities equipment operating and related, n.e.c.	14 (2027)	1.21 (0.72–2.05)	0 (115)	—
*Occupation not elsewhere classified*				
Laborers, n.e.c.	235 (59,634)	**1.19 (1.03–1.36)**	66 (15,018)	**1.38 (1.08–1.78)**
Other occupations, n.e.c.	33 (5297)	1.06 (0.75–1.50)	<6 (641)	—

*Note:* Statistically significant increased risks are bolded, statistically significant decreased risks are italicized.

Abbreviations: CI, confidence interval; HR, hazard ratio; n.e.c., not elsewhere classified.

^a^ Adjusted for age at start of follow‐up and birth year.

Several specific occupational groups, such as shipping and receiving clerks; driver‐salesmen; mining and quarrying cutting, handling, and loading; excavating, grading, paving, and related; among others, also demonstrated higher risks among males only. A reduced risk was observed among males employed as police and detectives. Across many of these specific occupational groups, females could not be assessed due to low case counts. Where reportable, females demonstrated higher risks than males, such as those employed as tellers and cashiers; and in food and beverage preparation and related service; laboring and other elemental work in chemicals, petroleum, rubber, plastic, related materials processing; electrical equipment fabricating and assembling, among others. A reduced risk was observed among females employed as nurses (registered, graduate, and nurses‐in‐training) (Table 3).

## 4 Discussion

This study used a unique cohort established through the linkage of administrative data to identify diverse groups at elevated risk of RP. This study had the ability to examine risk by both occupation and sex. Potential workplace exposures may explain some of the associations observed in this study. However, it is important to note that this study does not have specific exposure information but employs the occupational information as a surrogate for potential exposures.

Occupations with high exposure to vibration have been previously associated with the development of SRP. This includes occupations in which workers operate vibrating machinery and tools such as chainsaws, drills [9, 10], forklifts, locomotives, and tractors [47]. Exposure to vibration is likely among occupations where increased risks were recognized, such as workers in laboring and elemental work, machinist and machine tool setting‐up, truck drivers, and equipment operating. Other occupations (e.g., mining and quarrying; excavating and grading; clay, glass, and stone processing and machining) may expose workers to higher levels of vibration [14, 48]. Our study highlighted increased risks in some occupations which involve driving a motorized vehicle, such as truck drivers and driver‐salesman. Truck drivers experience increased levels of whole‐body vibration (WBV) which is transmitted through the seat, feet and steering wheel [7, 49]. HAV from the steering wheel of other types of vehicles such as tractors, has been associated with RP [50]. WBV and foot‐transmitted vibration (FTV) have also been associated with RP, but specifically in the feet [7, 23]. Although our study found increased risks in mining and quarrying, and specifically in cutting and handling, the number of cases was lower than expected as this sector is typically associated with SRP [51]. Upon further examination, we discovered that almost 80% (*n* = 1505) of WSIB compensated claims for RP in our cohort came from the mining and quarrying sector. These claimants were removed as they were considered prevalent cases of RP, resulting in a lower‐than‐expected risk among the remaining mining and quarrying workers in the cohort.

Cold temperature is a known trigger and a potential cause of RP through cold‐related injuries [25, 33]. There were various occupations (e.g., food and beverage processing; slaughtering and meat cutting, canning, curing, and packaging) in our study which may expose workers to cold working environments or localized cold exposure. Other studies have found similar findings with cold temperature‐related health risks for workers in the frozen food industry [52, 53]. Beyond food processing, other occupations such as mining, may have a high risk of cold injury [54]. Similarly, a study by Sinks et al [55] in Ohio using workers’ claim data found most occupational cold injuries occurred in oil and gas extraction and transportation, among other occupations. Workers, such as those in mining, who are exposed to multiple potential risk factors (e.g., cold and vibration) may be at greater risk than those only exposed to a single factor [31].

Occupational chemical exposures are less recognized as risk factors for SRP. Past studies have shown some evidence linking vinyl chloride to the development of SRP [16, 34], though regulations have reduced worker exposure [38]. We observed elevated risks among workers in laboring and other elemental work related to chemicals, petroleum, rubber, plastic, and materials processing, where vinyl chloride exposure is possible [56]. Additionally, organic solvents have also been associated with the development of scleroderma (leading to SRP) [35, 36, 57] and with SRP [18]. This may be a risk in specific occupations such as printing press operators, where this exposure is possible [17].

Overall, while many of the high‐risk occupations have known potential workplace exposures that are related to RP, for some occupations, such as sales or clerical and related occupations the potential exposures are not as evident. Further, some occupations where elevated risks were expected did not show associations (e.g. crushing and grinding or other plastic processing occupations). Various occupational groups had different sample sizes and at times case counts were too low to further analyze.

Notable differences between males and females were observed in this study. The overall incidence of RP in the cohort was higher for males, when compared to females, potentially highlighting sex differences between PRP and SRP, although we could not differentiate between the two types. The differing number of workers in each occupation may affect the results, for example in occupations where there were more males than females. However, sex differences were noted in various occupations that had similar case counts, such as sales; baking, confectionary making and related; and electrical equipment fabricating and assembling. Certain factors may contribute to the varying risks of RP between sexes. A study by Roquelaure et al. [25] focused on the risk of occupation‐related RP and found that cold exposure and smoking status were more influential in determining the risk for males, while psychological stress, high repetitiveness of tasks, insufficient recovery time, age over 45 years, and inadequate supervisor support were more influential for risk among females [25]. Another study by Harada et al. [58] reported vibration as the primary cause of RP for males. For females, it has been hypothesized that there is a biologically increased risk due to sex hormones. Several studies have explored the relationship between estrogen and the central and peripheral thermoreceptors, suggesting that fluctuations in the menstrual cycle could influence the impact of cold on digital blood flow [59, 60, 61]. Based on the findings of our study, males may be at a greater risk for occupation‐related SRP, however, there may be a combination of risk factors at play for both sexes.

A major strength of this study is the use of a large, linked cohort of workers, providing enough statistical power to examine risk of RP across many occupational groups and by sex. This study strengthens the existing evidence by supporting associations between occupational groups and the risk of RP, while also highlighting the risks among males and, more specifically, females, who are often understudied.

There are also limitations to this study. First, the case definition includes outcomes other than RP based on the ICD code available in the physician billing records. However, the other diseases coded under ‘Other peripheral vascular disease’ (Buerger's disease, other arterial dissections and other peripheral vascular diseases, etc.) are less prevalent, or more commonly use other ICD codes, therefore it is likely that the majority of our cases are RP‐related [41, 42]. Additionally, as the other conditions are independent of occupation, they only contribute to the baseline hazard. We also specified that workers had to have two records of an RP diagnosis to be included in this study, which further strengthens the case definition. As a secondary analysis, we assessed the risk of RP using ICD‐10 code I73.0 identified in hospitalization and emergency department records. There were fewer cases reported in these records, however we did see similar high‐risk occupations identified, such as mining and quarrying, food and beverage processing, metal machining, and motor transport operating. This demonstrates consistency with our results using various administrative health databases and both ICD 9 and 10 codes. Furthermore, as information on occupation is reported at the time of the claim, there is no information on the duration of employment or changing of jobs which may result in some misclassification. However, we restricted to a 5‐year follow‐up period to examine RP diagnoses closer to claims records which would help eliminate potential job changes beyond the 5 years of follow‐up. Use of the 5‐year follow up period may fail to capture occupation‐related RP cases which result from very low exposures over longer periods of time. Similarly, we cannot guarantee occupational exposure based on job title, potentially resulting in some exposure misclassification. As we do not know what the exposures are, we also do not know what their interactions nor the impact on sex differences might be. However, much of our findings are in occupations with known RP‐related workplace exposures. Furthermore, our study lacked information on family history of RP. Typically, cases of PRP with a family history have an earlier onset of symptoms between the ages of 15–30 [62]. As the median age at RP diagnosis was 53 and 52 years for males and females in our study, it is unlikely that family history of RP had an effect on our findings. While stratifying by sex provides an understanding of potential sex differences, we had many occupational groups with low case counts that were unreportable, particularly among females. We were also unable to adjust for cigarette smoking which may be a confounder. Selection bias is also likely in this study as the cohort consists of only those with accepted lost time compensation claims, so the cohort may systematically differ from and may not represent the general Ontario population. This may affect our findings by sex, as females are underrepresented in workers' compensation claims data. They are less likely to report an injury, and reported injuries are primarily among those in service and administrative occupations [63, 64]. On the other hand, males represent the majority of high‐risk occupations in compensation claims data [65]. We also acknowledge that we are making multiple comparisons which may lead to some chance findings. Finally, the HRs from Cox regression must be interpreted with caution as they are not a direct measure of attributable risk.

## 5 Conclusion

Overall, males and females showed similar elevated risks across various occupations but also demonstrated notable differences. These differences may stem from varying susceptibility and potential differences in exposure between the sexes, although this study was based only on job title, without data on exposures. Understanding the occupational groups with a higher risk of RP is crucial for targeting specific work environments to raise awareness of RP, reduce potential risk factors, and enhance overall workplace health and safety.

## Author Contributions

Ryann E. Yeo contributed to writing the original draft manuscript, review and editing, data analysis, formal analysis, methodology, and software. Fanni R. Eros contributed to supervision, writing the original draft manuscript, review and editing, data analysis, formal analysis, methodology, and software. Paul A. Demers contributed to review and editing, conceptualization, funding acquisition, and methodology. Jeavana Sritharan contributed to supervision, writing the original draft manuscript, review and editing, conceptualization, project administration, funding acquisition, and methodology.

## Disclosure by AJIM Editor of Record

John Meyer declares that he has no conflict of interest in the review and publication decision regarding this article.

## Ethics Statement

The study was conducted at the Occupational Cancer Research Centre, Ontario Health and was approved by the University of Toronto Ethics Review Board (#39013).

## Conflicts of Interest

The authors declare no conflicts of interest.

## Disclaimers

The authors have nothing to report.

## Data Availability

The data that support the findings of this study are available from Ontario Health. Restrictions apply to the availability of these data, which were used under license for this study. Data are available from the author(s) with the permission of Ontario Health.
